# Research ethics committees and regulation of climate change and health research

**DOI:** 10.2471/BLT.25.294147

**Published:** 2026-01-22

**Authors:** Godwin Pancras, Winfred Nazziwa, David Nderitu, Jovine Bachwenkizi, Renatha Joseph

**Affiliations:** a Department of Bioethics and Health Professionalism, Muhimbili University of Health and Allied Sciences, P.O. Box 65001, Dar es Salaam, United Republic of Tanzania.; b Research Compliance and Quality Assurance Unit, Uganda National Council for Science and Technology, Kampala, Uganda.; c Department of Philosophy, History and Religious Studies, Egerton University, Nakuru, Kenya.; d Department of Environmental and Occupational Health, Muhimbili University of Health and Allied Sciences, Dar es Salaam, United Republic of Tanzania.

## Abstract

Research has become essential in addressing the effects of climate change on human health and that of the biosphere. However, the ethical regulation of such research remains insufficiently developed, particularly with the current consolidation of research ethics committees as the reference standard to review and approve of health research. Unlike human-centred health research, climate and health research extends beyond humans to include biotic and abiotic components. This aspect challenges the human-centred approach to ethics that has traditionally defined the role of research ethics committees. In this analysis, we seek to re-examine the role of these committees in guaranteeing the ethical review of climate change and health research and the possibility of extending beyond its limited, human-centred scope. We also discuss the ethical concerns and considerations from the global and African-centred perspective that research ethics committees should address. We recommend that institutions hosting these committees implement two initiatives. First, restructure research ethics committees to include experts in climate change and health, as well as activists and representatives of Indigenous communities who are knowledgeable about the links between health and climate. Second, support initiatives to build the capacity of committee members, for example by developing training curricula on climate change and health research. These curricula should aim to strengthen the ability of committees to identify and address key issues including justice, intergenerational ethics and community-specific norms and values.

## Résumé

Il est devenu essentiel de mener des recherches pour faire face aux effets du changement climatique sur la santé humaine et la biosphère. Cependant, les règles d’éthique des études sur ces sujets restent insuffisamment élaborées, en particulier alors que les comités d’éthique dans la recherche se consolident comme norme de référence pour examiner et approuver la recherche médicale. Contrairement à la recherche médicale centrée uniquement sur l’être humain, la recherche qui allie changement climatique et santé inclut également des composantes biotiques et abiotiques. Cet aspect remet en question l’approche éthique centrée sur l’humain qui a traditionnellement défini le rôle des comités d’éthique. La présente analyse cherche à réexaminer le rôle de ces comités pour garantir l’aspect éthique de la recherche sur le changement climatique et la santé, ainsi que la possibilité d’étendre leur champ d’action au-delà de leur portée anthropocentrée. Elle adopte également un point de vue mondial et africain pour aborder des préoccupations et considérations éthiques, que les comités d’éthique de la recherche devraient prendre en compte. Enfin, elle recommande aux institutions qui hébergent ces comités de mettre en œuvre deux initiatives. Premièrement, restructurer les comités d’éthique dans la recherche afin d’y inclure des experts en matière de changement climatique et de santé, ainsi que des militants et représentants de populations autochtones qui connaissent bien les liens entre ces deux domaines. Deuxièmement, soutenir des initiatives visant à renforcer les capacités des membres de ces comités, par exemple en élaborant des programmes de formation sur la recherche en matière de changement climatique et de santé. Ces programmes doivent viser à renforcer la capacité des comités à identifier et à traiter des questions clés telles que la justice, l’éthique intergénérationnelle et les normes et valeurs spécifiques à chaque groupe de population.

## Resumen

La investigación se ha vuelto esencial para abordar los efectos del cambio climático en la salud humana y en la biosfera. Sin embargo, la regulación ética de este tipo de investigación sigue estando insuficientemente desarrollada, en particular en el contexto de la actual consolidación de los comités de ética de la investigación como el estándar de referencia para revisar y aprobar la investigación sanitaria. A diferencia de la investigación sanitaria centrada en los seres humanos, la investigación sobre el cambio climático y la salud se extiende más allá de las personas e incluye componentes bióticos y abióticos. Este aspecto cuestiona el enfoque ético centrado en el ser humano que tradicionalmente ha definido la función de los comités de ética de la investigación. En este análisis, se reexamina la función de estos comités para garantizar la evaluación ética de la investigación sobre el cambio climático y la salud, así como la posibilidad de ampliar su alcance más allá de su ámbito limitado y centrado en el ser humano. Asimismo, se analizan las preocupaciones y consideraciones éticas, desde una perspectiva global y centrada en África, que los comités de ética de la investigación deberían abordar. Se recomienda que las instituciones que albergan estos comités implementen dos iniciativas. En primer lugar, reestructurar los comités de ética de la investigación para incluir expertos en el cambio climático y la salud, así como activistas y representantes de comunidades indígenas con conocimientos sobre los vínculos entre la salud y el clima. En segundo lugar, apoyar iniciativas para fortalecer la capacidad de los miembros de los comités, por ejemplo mediante el desarrollo de planes de formación sobre la investigación en el cambio climático y la salud. Estos programas formativos deberían tener como objetivo reforzar la capacidad de los comités para identificar y abordar cuestiones clave, como la justicia, la ética intergeneracional y las normas y los valores específicos de las comunidades.

## ملخص

أصبحت الأبحاث ضرورية لمعالجة آثار تغير المناخ على صحة الإنسان والمحيط الحيوي. ومع ذلك، لا يزال التنظيم الأخلاقي لهذا النوع من الأبحاث غير متطور بشكل كافٍ، لا سيما مع ترسيخ لجان أخلاقيات الأبحاث كمعيار مرجعي لمراجعة واعتماد أبحاث الصحة. على عكس أبحاث الصحة التي تركز على الإنسان، تتجاوز أبحاث المناخ والصحة نطاق الإنسان لتشمل المكونات الحيوية وغير الحيوية. يُمثل هذا الجانب تحديًا للنهج الأخلاقي الذي يركز على الإنسان، والذي حدد بشكل تقليدي دور لجان أخلاقيات الأبحاث. في هذا التحليل، نحن نسعى إلى إعادة النظر في دور هذه اللجان في سبيل ضمان المراجعة الأخلاقية لأبحاث تغير المناخ والصحة، وإمكانية توسيع نطاقها ليشمل ما هو أبعد من نطاقها المحدود الذي يركز على الإنسان. كما نناقش المخاوف والاعتبارات الأخلاقية من منظور عالمي وأفريقي ينبغي على لجان أخلاقيات البحث معالجتها. نحن نوصي المؤسسات التي تستضيف هذه اللجان بتنفيذ مبادرتين. أولًا، إعادة هيكلة لجان أخلاقيات الأبحاث لتشمل خبراء في تغير المناخ والصحة، بالإضافة إلى ناشطين وممثلين عن مجتمعات السكان الأصليين ممن لديهم معرفة بالروابط بين الصحة والمناخ. ثانيًا، دعم مبادرات بناء قدرات أعضاء اللجان، على سبيل المثال من خلال تطوير مناهج تدريبية حول تغير المناخ وأبحاث الصحة. ينبغي أن تهدف هذه المناهج إلى تعزيز قدرة اللجان على تحديد ومعالجة القضايا الرئيسية، بما في ذلك العدالة، والأخلاقيات بين الأجيال، والمعايير والقيم الخاصة بكل مجتمع.

## 摘要

开展研究已成为应对气候变化对人类健康和生物圈的影响的至关重要手段。但是，此类研究的伦理监管框架仍不够完善，特别是在当前强化研究伦理委员会的地位并将其视为审查和批准健康研究的参考标准的情况下。与以人为中心的健康研究不同的是，气候和健康研究所涉对象超出了人类范畴，需将生物和非生物元素全部考虑在内。这一转变将对以人为中心的伦理学方法构成挑战，而这种方法又是一直以来界定研究伦理委员会角色的基础。在本文的分析部分，我们试图再次研究这些委员会在保障气候变化和健康研究的伦理审查方面所扮演的角色，以及将研究对象扩展至以人为中心的限定范畴以外的可能性。我们还从全球和以非洲为中心的视角讨论了研究伦理委员会应应对的伦理问题和考量因素。我们建议负责成立这些委员会的机构推行两大举措。首先，重新组建研究伦理委员会，将气候变化和健康领域的专家以及了解健康和气候之间关联性的土著社区活跃分子和代表纳为委员会成员。其次，支持推行旨在增强委员会成员能力的举措，例如安排气候变化和健康研究相关培训课程。这些课程应着眼于强化委员会识别和解决包括公正、代际伦理及社区特定规范和价值观在内的关键问题的能力。

## Резюме

Исследования стали неотъемлемой частью решения проблем, связанных с воздействием изменения климата на здоровье человека и биосферу. Однако этическое регулирование таких исследований остается недостаточно развитым, особенно в условиях нынешнего укрепления роли комитетов по этике исследований как эталонного механизма рассмотрения и одобрения исследований в сфере здравоохранения. В отличие от исследований, ориентированных исключительно на человека, исследования в области климата и здоровья выходят за рамки исключительно человеческого измерения и охватывают как биотические, так и абиотические компоненты. Этот аспект ставит под вопрос человекоцентричный подход к этике, который традиционно определял роль комитетов по этике исследований. В представленном анализе авторы хотели бы пересмотреть роль этих комитетов в обеспечении этической экспертизы исследований в области изменения климата и здоровья, а также возможность выхода за пределы ограниченной человекоцентричной точки зрения. Авторы также обсуждают этические проблемы и соображения с глобальной и афроцентричной точек зрения, на которые этическим комитетам исследований стоит обратить внимание. Учреждениям, в рамках которых работают такие комитеты, можно рекомендовать для внедрения две инициативы. Во-первых, изменить структуру комитетов по этике, введя в их состав экспертов в области изменения климата и здоровья, а также активистов и представителей коренных сообществ, которые обладают знаниями о связях между климатом и здоровьем. Во-вторых, поддержать инициативу по наращиванию потенциала членов комитета, например путем разработки учебных программ, посвященных исследованиям в области изменения климата и здоровья. Эти программы должны быть направлены на укрепление способности комитетов выявлять и учитывать ключевые вопросы, включая принципы справедливости, межпоколенческую этику, а также специфические для сообществ нормы и ценности.

## Introduction

The promotion of ethically sound research and the avoidance of extractive research practices depend on the existence of good research regulatory mechanisms. Despite the increasing interest in climate and health research, the role of existing research ethics committees or institutional review boards remains less examined. Historically, research ethics committees were established as a response to unacceptable and inhumane experiments involving vulnerable individuals.^1^^–^^3^ However, climate change and health research present a new moral dimension that challenges the status quo of how research ethics committees ought to function. The key function of any of these committees is to ensure the protection of human rights and the well-being of research subjects.^4^ However, this conception presents philosophical challenges regarding climate change and health research regulation.

From a philosophical perspective, research ethics committees could be viewed as being more anthropocentric (human-centred) than biocentric (all life forms) or ecocentric (ecosystem) due to the assigning of moral values exclusively to humans,^5^ while the environment or other species are seen as mere means to achieve human needs.^6^ This traditional lens could translate to an outdated perspective that is human centred. While this approach may be defensible for human research, it may be questionable for climate change and health research, where the concept of research subjects extends beyond humans to encompass other biotic (living things) and abiotic (non-living things) components. Climate change and health research can yield considerable human benefits, but at the expense of the environment or other species. For example, solar energy research may lead to large solar farms that reduce emissions but also disrupt wildlife habitats; biofuel research can improve energy security while driving deforestation and biodiversity loss; and climate-resilient agriculture can enhance food security while reducing genetic diversity or affecting native species. Large-scale geoengineering proposals could help cool the planet, but they also create ecological risks for other living beings. Balancing these trade-offs requires rigorous environmental impact assessments, ecosystem-based approaches and ethical research governance frameworks that consider non-human interests.

This alteration from anthropocentrism as a central tenet of research ethics committees to ecocentrism warrants a critical examination to uphold the ethical standards in climate change and health research. Nonetheless, the fundamental question remains as to whether the role of research ethics committees could be extended beyond human health and, if so, what ethical concerns should be considered. 

## Moving to planetary health ethics

The need to protect the environment and ensure the flourishing of all life forms is defined by the term bioethics, coined in 1927.^7^ This approach posits that every living being should be respected as an end in itself and treated accordingly.^8^ The acts committed by Nazi doctors during the Second World War and technological advancement in medicine revitalized the need for bioethics. The acts of Nazi doctors included coercing people into inhumane experiments involving deliberate hypothermia, exposure to mustard gas and head injury, among others.^9^^,^^10^ Other unethical experiments include the Tuskegee syphilis study, Willowbrook state school hepatitis study and many more.^2^ During this period, bioethics became focused on the well-being of humans participating in research.

In the early 1970s, academic institutions adopted and institutionalized the human-centred view of bioethics with a focus on medical ethics.^11^^,^^12^ Medical ethics concern the dilemmas in physician-patient or researcher-participant relationships, that is, the human-to-human interaction. Similarly, despite evidence of the human assault against the environment since the 1940s,^13^ environmental ethics as an academic discipline did not gain prominence until the 1970s.^14^ To date, environmental ethics lag far behind that of medical ethics as shown by the limited number of guidelines, academic programmes and empirically supported scholarly writings in environmental ethics. For example, while provisions referring to environmental protection during research are non-existent in the current *International ethical guidelines for health-related research involving humans*,^15^ the recent amendment to the *Declaration of Helsinki* only briefly mentions environmental protection, with no clear guidance.^16^ These documents shape the global research regulatory landscape. However, given the increasing concern for human and non-human health, some scholars have called to move beyond environmental ethics, which champions sustainability, to planetary health ethics, which insists on urgency, advocacy, interconnectedness and inclusivity in addressing climate change ethical issues.^17^ The richness and broadness of the planetary health approach make it possible to merge global health, Eco Health and One Health perspectives.^18^^,^^19^ Therefore, it would be logical that the ethics principles of planetary health, and not traditional bioethics, inform the regulation of climate change and health research. 

## Expanding the current paradigm

Existing ethics guidelines have defined the scope of the committees’ role within the limits of the fundamental research ethical principles, that is, respect for persons, beneficence and justice.^20^ The purpose of the fundamental principles is only to protect the rights of human beings participating in research. Nevertheless, in the context of climate change and health research, the question is whether the scope of this role should be extended, or a different committee should be established to review climate and health research. Here, we argue for the former and against the latter. Establishing a different entity may strain the already limited human resources of skilled workers and add a logistical burden, particularly in low-income countries where some research institutions may not have research ethics committees or may face financial constraints, limited office spaces and fewer skilled members.^21^^,^^22^ Moreover, since these committees are the regulatory agents of bioethics research, extending their role to encompass the well-being of all life forms would reintroduce the concept of bio to ethics.

Extending the review paradigm of research ethics committees is feasible. The extension could take advantage of the nature and structures of existing committees. These committees have the intrinsic objective to protect the fundamental rights of human participants, which could be extended to the protection of the rights of nature. These rights involve the legal movement advocating for humans to recognize the value and rights of non-humans and the ecosystem.^23^ Moreover, such an extension would benefit from existing international policies ingrained in the *Convention on biological diversity* and the proposed *Universal declaration for the rights of mother earth*, among others. Climate change and health research regulation could take advantage of the structural organization of research ethics committees, from membership composition to research management structures, to strengthen this extended role. For example, traditional research ethics committees already have mechanisms to review and approve protocols, receive and resolve research disputes, and the means to conduct passive or active oversight. These mechanisms are equally important in ensuring that climate change and health research is conducted within the purview of ethics. However, in so doing, this paradigm shift must appreciate and operationalize existing global and African-centred views that could play a crucial role in regulating climate change and health research. 

## Core ethical concerns 

The concerns include but are not limited to justice, intergenerational ethics, solidarity and equitable partnerships.^24^ However, here we pay attention to justice, intergenerational ethics and African-centred views that research ethics committee ought to appreciate as they assume the regulatory role of climate change and health research. 

### Upholding justice 

According to the sociological model of climate justice,^25^ justice can be grouped into four elements: (i) distributive or material justice, focusing on fair distribution of services or goods and burdens; (ii) procedural justice, focusing on the fairness of the decision-making process; (iii) compensatory justice focusing on rectifying injustices either through compensation or reparations; and (iv) transformative justice, advocating for systemic changes and addressing underlying injustices.^25^ In this article, we consider the principles of distributive justice and procedural justice to be of immediate concern in climate change and health research, since the other principles (compensatory and transformative justice) may require legal instruments beyond the purview of research ethics committees. To uphold the principle of distributive justice in climate change and health research, committees need to establish reasonable mechanisms to evaluate study benefits or co-benefits and risks beyond human beings. For benefits, committees must discern who benefits, what are the potential benefits and their durability and affordability. As to risks or burdens, committees should discern the likelihood and magnitude of the risks, who is likely to be burdened the most and whether the risks can be alleviated or minimized. Moreover, committees ought to consider the risk-benefit trade-offs whereby the risks may be immediate but the benefits could be enjoyed in the future, probably, not by the people or ecological communities (humans and non-humans) that participated in the study. 

Procedural justice offers an ethical lens through which fairness can be integrated into the distribution of benefits and risks of climate change and health research, mostly by emphasizing an inclusive decision-making approach.^26^ To uphold procedural justice, research ethics committees would be obliged to ensure that all parties affected by the proposed study have an equal opportunity and power to influence the decision-making process. Since the effects of climate change weigh heavily on the most vulnerable, the consequences of leaving them out in decision-making processes cannot be overstated. Building on the rules of community engagement in human-centred research, research ethics committees can instruct researchers to describe how they plan to engage different stakeholders in decision-making, specifically how Indigenous communities and other vulnerable populations will be empowered to make decisions. Research ethics committees should consider that the decision-making process will mostly involve negotiations. Negotiations are rarely value-free, and one party may resort to unethical or inappropriate tactics to persuade the other side to reach an agreement.^27^ Given such circumstances, committees may provide parties with guidance on how to ethically conduct their negotiations for a more equitable decision-making outcome.

### Intergenerational ethics 

In addition to justice, intergenerational ethics is becoming an indispensable component of the ethics of climate change and health research. Scholars agree that the discussion about climate change is inherently a discussion about intergenerational responsibility or equity.^24^^,^^28^ The concept of intergeneration is grounded in the idea that each generation should leave the Earth and its natural and cultural resources at least as they received them. In human-centred studies, it is considered unethical when research participants are left in a worse-off state at the end of their participation compared to when they started.^29^ However, in climate change and health research, intergenerational ethics would inquire into the risk–benefit analysis between current and future generations, with research ethics committees as the stewards of that analysis. This responsibility is further complicated by a growing reliance on technological fixes and by the use of research to legitimize innovations that are presented as solutions to the climate crisis. Thus, research ethics committees may have limited capacity to foresee whether the outcome of such technologies or innovations would worsen or improve the health of future generations. However, this dilemma may be resolved if committees pay more attention to and apply the three principles ingrained in intergenerational equity: conservation of options, quality and access.^30^ For options, committees should analyse the research’s ability to conserve the diversity or plurality of natural and cultural resources. For quality, committees should ensure that the quality of the ecosystem in which the research is to be conducted is not left in a worse-off condition for the current or future inhabitants. Lastly, committees can uphold the principle of access by ensuring that the outcome of the proposed research does not exacerbate the inequalities and discrimination among and between generations.

### African-centred views 

Research is conducted in specific contexts, with underlying cultural norms, practices and belief systems. These systems have implications on how research is received, accepted or appreciated, depending on how it respects local perspectives. The research community has often expected research ethics committees to ensure that local contexts are considered and respected as an ethical ideal during reviews and approval of protocols. This responsibility explains why the committees’ membership must include a community representative or lay person whose role includes, among others, ensuring that the research is culturally sensitive and the local context is well understood.^31^

In addition to the global ethical imperatives, research ethics committees should appreciate African cultural perspectives and values when reviewing climate and health research from the continent. *Ubuntu* is often touted as the foundational cultural philosophy common in many African communities. This philosophy is meant to promote the welfare of humanity and to inspire people to take care of each other for the sake of the present life and future generations.^32^ Nonetheless, the welfare that *Ubuntu* intends is often extended beyond mere human relationships to include all forms of existence, as seen in the African Indigenous ontology of nature. In the African ontology of nature,^32^^,^^33^ life is perceived as an interconnected system of hierarchical categories of existence, from God, spirits and divinities, human beings and animate nature to inanimate things. This conceptualization of nature holds that every individual member of society must take care of the environment because a human being is inseparable from the environment.^34^

Values and norms that prescribe correct behaviour towards nature have long existed in Africa.^35^ For example, the Tonga people of southern Zambia employ selective harvesting, totemism and taboos, organic farming, crop rotation and intercropping, the sacredness of water sources and traditional authority to sustain their local biophysical environment, including conservation of soil, water, animals, medicinal and fruit plants and rangeland.^36^ The Gedeo community of southern Ethiopia uses the Songo Indigenous institutions, traditional beliefs, taboos and local rules to promote environmental protection and cultural conservation. The setting aside of sacred forests for ritual purposes is an Indigenous mechanism of tree biodiversity conservation. Social taboos, like Indigenous belief systems, have limited people from cutting down trees in sacred sites, carelessly killing birds and injuring nature.^37^ The Kipsigis community in Kenya has had long-standing cultural practices that enhanced the conservation of the biosphere, especially by taking care of the Mara river basin. This community also observed some taboos in aid of environmental conservation; for example, cutting trees along the rivers and springs was not allowed.

Perhaps the environmental ethics of the Kom community is touted as the archetype for Indigenous approaches to the preservation of the biosphere that can be leveraged in the efforts to contribute to climate and health research regulation in Africa. Kom environmentalism is holistic and includes humans, the ecosystem, spirits, the living dead, as well as the unborn in the moral community.^35^ This reflects the approach of many African beliefs regarding the environment that is built upon the foundation of *Ubuntu*. Moreover, this worldview is reflected in Kom environmentalism, where no clear separation exists between humans and the rest of nature. In this belief, human beings are prohibited from interfering with the harmony that keeps nature intact. Some African scholars^32^^,^^38^ consider human beings as the only beings that have the capacity to enhance but also disrupt harmony in the universe. Most communities in Africa have moral codes and practices that regulate interhuman and interspecies relationships.^35^ The predominant environmental ethics in Africa, based on the interconnectedness of the universe, leans towards ecocentrism more than anthropocentrism.

Given the holistic worldview of the Kom ethics, regulations are needed that guide research practices in ways that respect African environmental values across the entire ecosystem. Some of these values and beliefs held by the African communities might be broadly similar to other contexts. For example, Indigenous Australian communities such as the Aboriginal people and Torres Strait Islanders consider human beings and nature as inseparable, living in a balanced harmonious Country,^39^^,^^40^ as do Native Americans.^41^ However, the values and beliefs regarding the human-nature interaction may not always be homogenous across communities. 

## Conclusion and recommendations

Discussions on climate change and its effect on health have led to an intensification of research in this domain. However, scholars have given comparatively little attention to the effectiveness of regulatory and oversight mechanisms for climate change and health research, particularly in light of its broader environmental and ecological dimensions. Globally, the rationalization of research ethics committees is built upon their traditional role of protecting human participants in research. Yet climate change and health research introduce aspects that encompass the entire ecosystem. Moreover, for low- and middle-income countries, particularly in Africa, contextual realities complicate the role of the committees in reviewing and regulating research on climate change and health. These challenges include limited institutional capacity and mechanisms for comprehensive oversight, and the presence of traditional values and norms that introduce unique perspectives to review processes. Because of these gaps, research ethics committees in many low- and middle-income countries are likely to have difficulties in reviewing and monitoring research protocols related to climate change and health, thereby exacerbating the risks to human participants and the entire ecosphere. This situation would also result in an unjust distribution of the benefits and burdens of climate change and health research. We therefore recommend that institutions hosting research ethics committees implement two initiatives. First, restructuring of research ethics committees to include experts in climate change and health, including activists and representatives from Indigenous communities knowledgeable about the interconnectedness between health and climate. These representatives could be identified from established nongovernmental organizations that are trusted by the community. In addition to an advisory role, experts should also have voting powers during committee proceedings to uphold procedural justice. Second, supporting initiatives to increase the capacity of research ethics committee members, through for instance developing training curricula that address issues of climate change and health research. The curricula should aim to develop the skills of research ethics committees in recognizing and addressing pertinent issues in climate change and health, including but not limited to justice, intergenerational ethics and community-specific norms and values.
